# Scholastic status of congenitally blind children following sight surgery

**DOI:** 10.52291/ijse.2022.37.49

**Published:** 2023-01-09

**Authors:** Shakila Bi, Ajay Chawariya, Suma Ganesh, Priti Gupta, Youqi Huang, Kimiya Jazayeri, Rakesh Kumar, Chetan Ralekar, Chaitanya Singh, Adya Tiwary, Lukas Vogelsang, Marin Vogelsang, Mrinalini Yadav, Pawan Sinha

**Affiliations:** 1 Dr. Shroff’s Charity Eye Hospital, New Delhi, India; 2 Indian Institute of Technology, New Delhi, India; 3 Lynbrook High School, San Jose, CA, United States; 4 Brookline High School, Brookline, MA, United States; 5 Massachusetts Institute of Technology, Cambridge, MA, United States; 6 Shiv Nadar High School, New Delhi, India; 7 Winston Churchill High School, Potomac, MD, United States; 8 École Polytechnique Fédérale de Lausanne, Lausanne, Switzerland

**Keywords:** Congenital blindness, special education, mathematics, diagnostic tests

## Abstract

India is home to a large population of blind children, many with treatable conditions. Project Prakash identifies treatable blind children and provides them with eye surgeries. Once treated, these children are given the opportunity to further their education. To understand their educational needs, we undertook a diagnostic screening exercise. Specifically, we designed math proficiency assessments and evaluated the scholastic preparation of 54 Prakash children across a broad age range. We found that the proficiency of most Prakash children was well below age-appropriate levels. Even those enrolled in high schools had assessed proficiencies around the 3rd-grade level. Furthermore, due to a lack of basic instruction on interpreting print material, many children continued using Braille even after gaining sight. The contrast that these findings present relative to the official standing of the children in their schools makes a compelling case for more rigorous assessments and better educational interventions for visually-impaired/sight-restored children. Also, we argue that these educational interventions should be coupled with visual function assessments to ensure that the presented material is accessible to the child. Our scholastic assessments, suitable for being administered over the phone, will be made available for use by other researchers and educationists.

## BACKGROUND

**Fig. 1.**
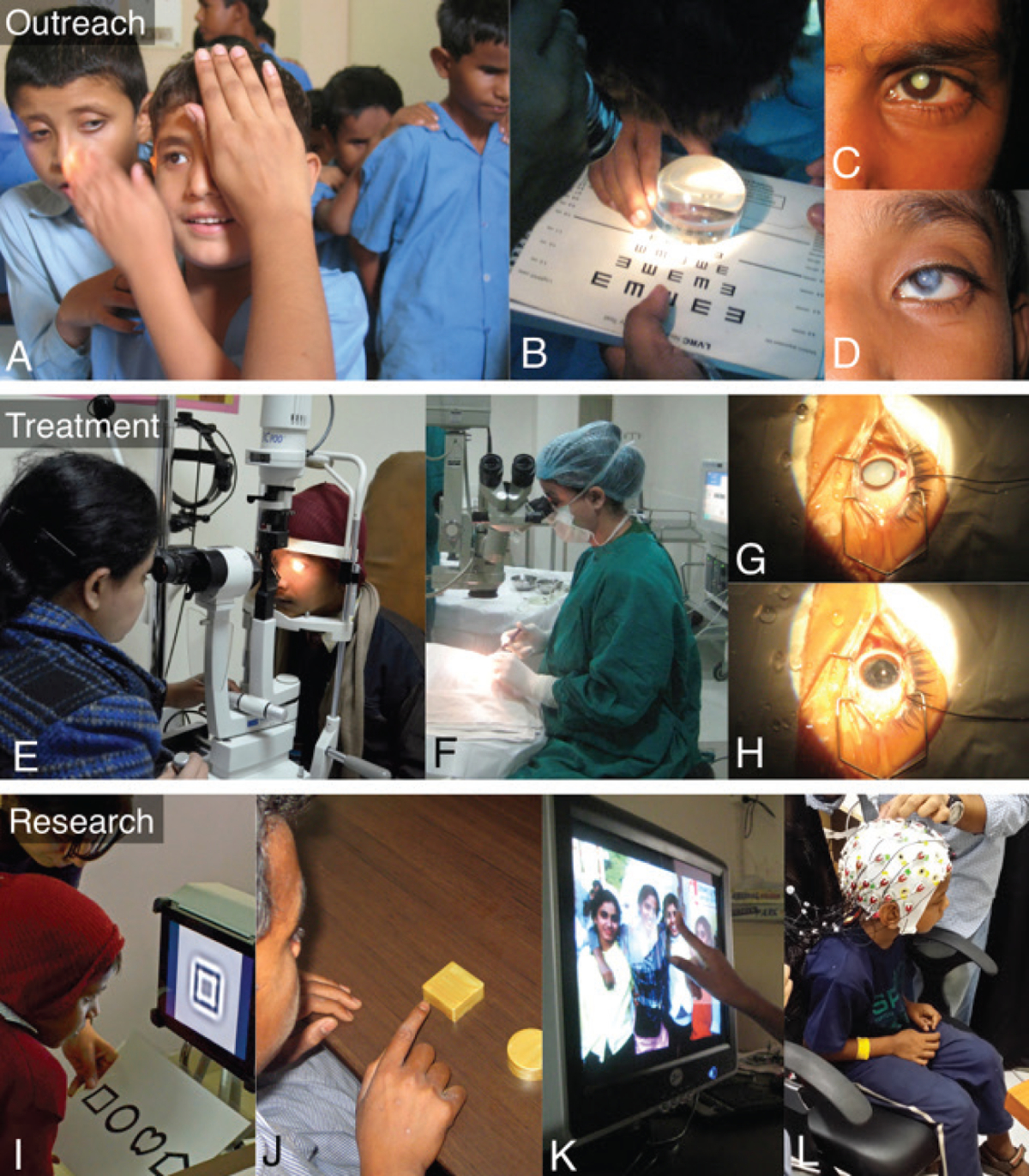
Project Prakash comprises three components: Outreach to identify children with treatable visual impairments and blindness (panels A-D); Treatment that typically involves pediatric cataract surgery (panels E-H); Scientific research to examine the development of vision following sight onset (panels I-L).

We seek to understand the educational status of an unusual population of children. These are children who were born blind in India and, due to financial or geographical reasons, stayed deprived of medical care, even though their blindness was treatable. Since 2005, an MIT-based initiative, Project Prakash, has actively searched for such children and provided them with eye surgeries (Sinha, 2013). In doing so, the project has attempted to address the pressing humanitarian need to treat blind children and has also gained scientifically valuable insights regarding how the brain adapts to the influx of visual information late in childhood. The work has positively impacted the quality of life of the treated children (Kalia et al., 2017) and has also uncovered several crucial results regarding the timelines and mechanisms of visual development (Gupta et al., 2022; Gandhi et al., 2017; Ostrovsky et al., 2009). Thus far, the project has brought sight to over 500 blind children. Figure 1 shows a few vignettes from Project Prakash.

We focus here on the educational trajectories of the treated children. In following up with the children, we found that even after gaining sight, several of them were unable to get admission into regular schools, which were unwilling to make accommodations for lingering visual deficits (such as sub-par visual acuity (Ganesh et al., 2014) and unfamiliarity with print). Hence, the Prakash children find it challenging to gain a foothold on the path that could lead them to eventual employment and financial independence. Deprived of opportunities for mainstream educational advancement, the children either continue at schools for the blind or, worse, are confined to home. Girls are often married off young, effectively extinguishing any possibility of their self-actualization.

Given the Prakash children’s near-total lack of mainstream educational options, there is a clear need to provide them with a ‘bridge’ course. The figurative ‘bridge’ is between their current scholastic preparation (acquired through their enrollment in schools for the blind) and a middle-school level of proficiency. After progressing through such a course, children will have a better chance of being mainstreamed into conventional schools since they would not require remedial accommodations from these schools.

To design such a bridge course, a necessary first step is to assess the current state of scholastic preparation of the Prakash children. This is the goal we focus on in this report. Specifically, within mathematics, we describe the diagnostic tests we prepared for a range of grades, the procedures we followed in administering them, and, importantly, the results of these assessments. We have chosen to constrain our initial investigation to mathematics since the subject is essential for many real-world tasks (e.g., maintaining home accounts and shopping) and critical for success in other areas, such as science. The findings are sobering, revealing the dismally low level of scholastic readiness of the Prakash children despite having been enrolled in schools for the blind for multiple years. The results are instructive not just for our specific goal of determining what level to target our bridge course but, more broadly, about the need to examine how the current schooling system for the blind must be improved to make a substantive impact on the intellectual preparation of its students.

### Past work

The newly sighted Prakash children constitute such a unique population that no prior work on their educational status exists in the literature. The reported data relate exclusively to untreated blind children. The findings, by and large, paint a disheartening picture. For most such children in India, education is a rare privilege. The paucity of schools catering to blind children is a significant bottleneck. Special schools for the blind can accommodate only about 8% of all blind children in the country (Rahi et al., 1995). The social stigma associated with the condition (Rohwerder, 2018) often induces parents to keep their blind children confined to home. As for the possibility of integrating severely visually impaired children in mainstream schools, the statistics are discouraging. According to a nationwide survey conducted in India by the state-run National Council for Educational Research and Training (NCERT), only 21.11% of all schools in India have provision for inclusive education for children with disabilities (Sreekanth, 2016). Even more sobering is the low rate of teacher training in special education. Only 1.32% of all teachers have any kind of training to work with children with disabilities. Given this lack of preparation of the schools and teachers, even for the children who gain access to a school, the actual acquisition of education is a challenge.

Mathematics education has been a particularly difficult challenge for the visually impaired (Bell & Silverman, 2019). The reliance on equations with special symbols not represented in Braille, as well as the reliance on graphs and other diagrammatic material, makes mathematical concepts hard to convey to blind students. This is true not just in the developing world but also globally. Blind students are typically unable to pursue interests in the sciences (Maguvhe, 2015; Ediyanto & Kawai, 2019), partly due to the need for a firm grounding in mathematics. Nemeth Code, abacus, and Braille writers have been used traditionally for blind children (Brawand & Johnson, 2016) to help them do simple calculations.

As students are progressing from primary school to higher grades, they must rely on computation aids to handle more complex concepts related to algorithm flow, sequencing, geometric forms, graphing, and measurements. Recent years have seen encouraging innovations, although they are yet to make their way to widespread deployment. For instance, process-driven mathematics, based on audio methods of instruction, has been developed for students who are no longer able to use traditional low-vision tools like Braille and Nemeth Code (Gulley et al., 2017). Maćkowski et al. (2018) have developed a multimedia-enabled platform for interactive learning of mathematics by the blind. Beal and Rosenblum (2018) found that applications on tablet computers are motivating ways to teach mathematics concepts, eliciting greater engagement from students relative to traditional literacy methods. Despite these positive recent developments, it is fair to say that the current state of math education for the blind leaves much to be desired, as is evident from the conspicuous absence of blind students in math and science programs in schools and colleges. It is not clear precisely when in the schooling trajectory this push away from mathematics commences. Do blind children perform on par with their sighted counterparts until the middle grades, before specialized symbols and equations begin to be used heavily? Answering this question requires appropriate diagnostic tests that objectively measure students’ math preparedness at different grade levels. However, designing such diagnostic tests has been a challenge as many concepts require visualization.

With this background, the two specific aims of the work we report here were:

- The design of diagnostic tests for a range of grades that could be administered to Prakash children over the telephone without the need for graphical communication
- The collection and analysis of data on diagnostic tests

## METHODS

### Participants

**Fig. 2.**
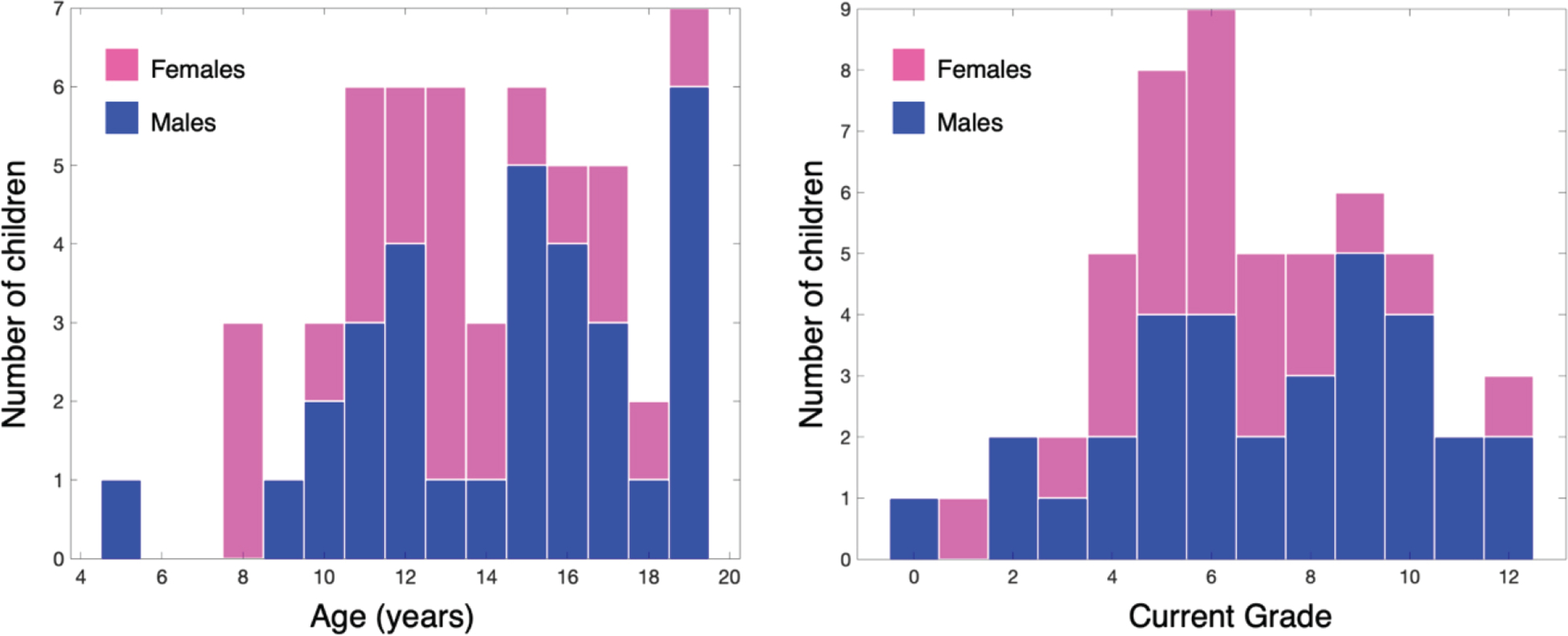
(a) Distribution of ages across our participant group, (b) The grades that students were enrolled in at the time of the study.

The children who participated in this study were all identified and treated as part of Project Prakash during the past 12 years. Initial identification took place at outreach eye screening camps in various states of north India. All children were diagnosed as having congenital bilateral cataracts. We contacted 75 Prakash patients (47 males, and 28 females). Of these, 17 were excluded since they were older than 20 years, and 4 had to opt out due to other obligations. Thus, our final participant pool comprised 54 children (32 males, and 22 females) ranging in age from 5 to 19 years (Figure 2a). There was no statistically significant difference in the ages of the male group relative to the female group (Male group: age range = [5, 19], mean = 14.5; Female group: age range = [8, 19], mean = 13; t(52) = −1.6, p = 0.12 in two-sample t-test). 48 children were provided free surgical treatment at Project Prakash’s medical partner institution, Dr. Shroff’s Charity Eye Hospital, New Delhi. Surgeries for the remaining six were delayed due to the pandemic but are expected to be conducted in the spring of 2022. The Prakash team stayed in touch with the children to monitor their visual status as well as their progress on social and educational dimensions. For the present study, the Prakash team commenced re-contacting these children in August 2021. We collected information about their general health and present educational status, i.e., whether they were enrolled in a school and, if so, in what grade.

#### Current enrollment status and grade level:

Out of the 54 participants, 50 were enrolled in a school. 4 children were not enrolled in a school at the time of the study but had passed different grades (one had passed 12^th^ grade, two had passed 10^th^ grade, and one had passed 8^th^ grade). Figure 2b shows the distribution of children across grades.

### Diagnostic Assessments

Based on the guidelines specified by NCERT and the Central Board for Secondary Education (CBSE), an Indian governmental body tasked with defining school curricula for the entire country, we formulated lesson plans and questions appropriate for grades one through five. For each grade, we identified the key topics the students are expected to understand (for example, first-grade topics included basic arithmetic operations, word problems, and applications such as time durations and carrying out a change with coin denominations). For each topic, we generated a series of questions spanning a range of difficulty levels (governed in part by the magnitude of the numbers involved in a problem; 3 + 4 being more accessible than 23 + 61) and ensured that they did not require any pictorial components like graphs or diagrams. From this pool of questions, we created two versions of each assessment, a short test for the initial assessment and a more extended test if gathering more information was deemed necessary by the proctor (as described in the next section). In developing these tests, we solicited input from two external elementary school educators at Florida Ruffin Ridley School (Brookline, MA). In the final step, a speaker fluent in English and Hindi translated the assessment material to Hindi allowing for it to be administered to children in north India. These steps were adopted for generating assessments for grades one through five.

### Administration of the diagnostic tests

Tests were administered telephonically by six Prakash team members fluent in Hindi. Children were intimated about the test a day before to ensure they were available and prepared. Most questions in the assessments were designed to require only mental computations. Still, the children were free to use any writing or Braille material they wished if doing so facilitated the calculations. Assessment of each child started at grade level 1. The shorter test was administered before proceeding to the longer one. The more extended test was rendered superfluous and was not administered if the participant could not answer more than half of the questions on the short assessment. If the child scored more than 80% on the long assessment of a given level, testing moved on to the next level. The last assessment level for which the child did not reach the passing score was taken to be his/her current proficiency. Each level’s assessment took about 20–30 minutes to administer over the phone.

## RESULTS

### Demographics

**Fig. 3.**
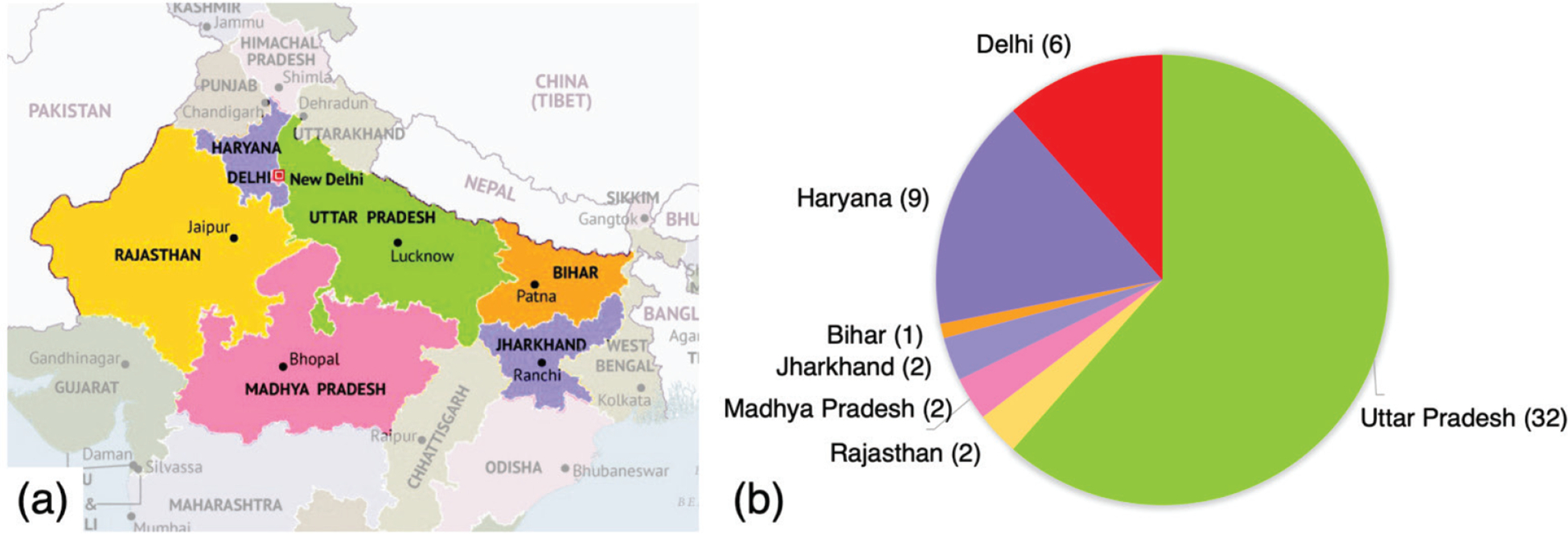
Geographical distribution of the children participating in the study. (a) Our participants came from seven north/central Indian states. (b) Number of participants from each state.

The children belonged to 7 states in north/central India. The distribution is shown in figure 3.

#### Socio-economic status:

All children in the participant pool came from families with household incomes lower than $150 per month. Since each household comprised three or more members, their income places them below the International Poverty Line (indicating ‘absolute’ or ‘extreme’ poverty), as defined by the World Bank (2016). None of the parents had had schooling beyond fifth grade, and most were illiterate. They worked as laborers on construction sites or farms. None of the children had access to personal computers or broadband internet.

## RESULTS OF ASSESSMENTS

### Mode of written communication

**Fig. 4.**
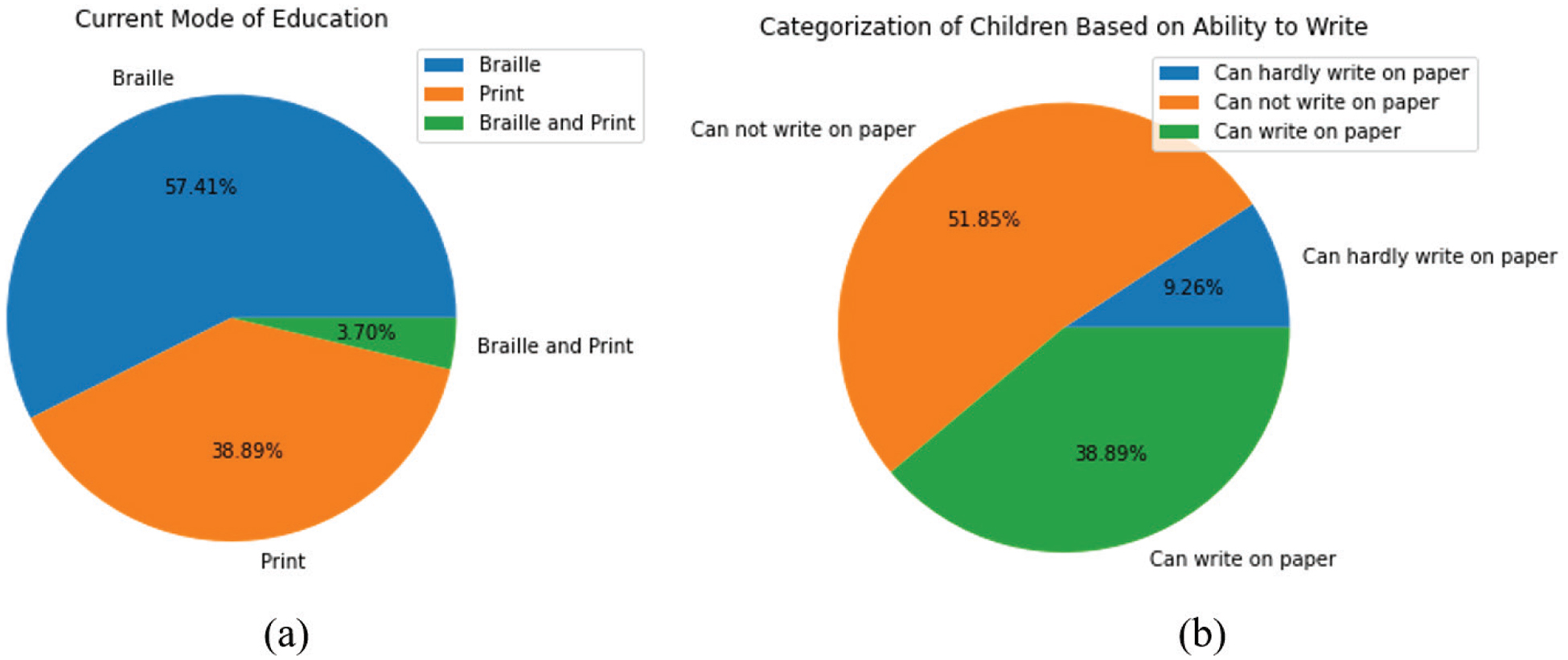
(a) Distribution of children based on their current mode of education. (b) Categorization of children based on their ability to write on paper.

Before their treatment, all children were enrolled in schools for the blind, where the mode of instruction was exclusively Braille. Following surgery, despite gaining adequate vision to work with enlarged text, only a minority of the children have shifted from Braille to print (figure 4a). This is because many children must continue in blind schools due to the reticence of regular schools to offer them admission.

Results on the non-Braille reading/writing ability of these children follow the general pattern depicted in figure 4a. As shown in figure 4b, 51.85 % of the children are unable to write on paper, 9.26 % have a very rudimentary ability to write, and 38.89 % can comfortably write on paper (this is the group that had transitioned to print as the mode of education).

#### Comparison between current and assessed grade

**Fig. 5.**
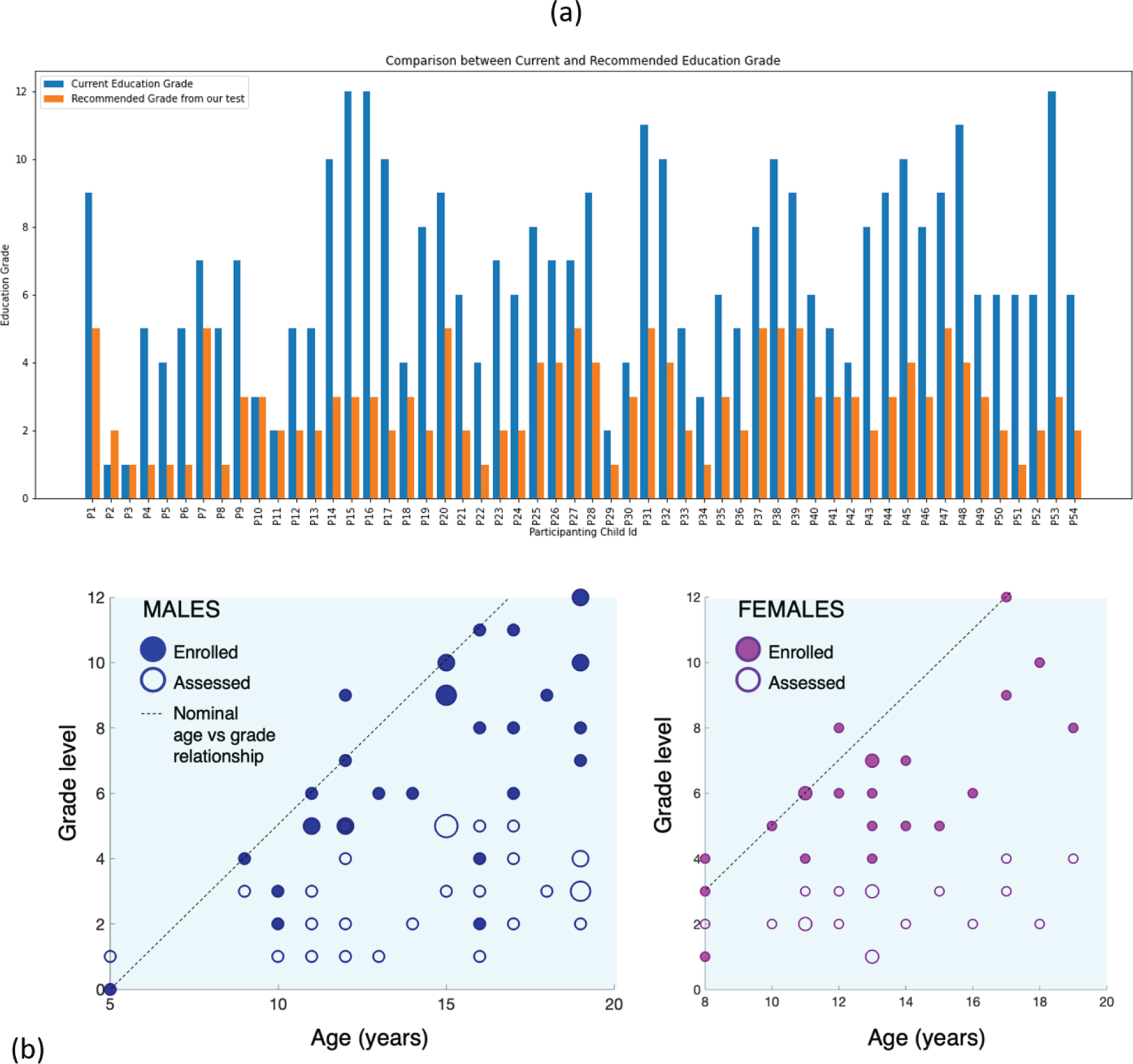
(a) Comparison between the current and recommended grade. (b) Scatterplots of enrolled and assessed math proficiency across ages for male and female Prakash children.

Figure 5a compares the recommended and current education levels for all participants. Figure 5b plots grade levels (in which the child is enrolled, as well as what the assessments reveal) across ages and separated by gender. Strikingly, most students performed well below their putative grade levels. This was especially notable in the case of children studying in high school, who were found to have a level of mathematical readiness below grade 4.

## DISCUSSION

**Fig. 6.**
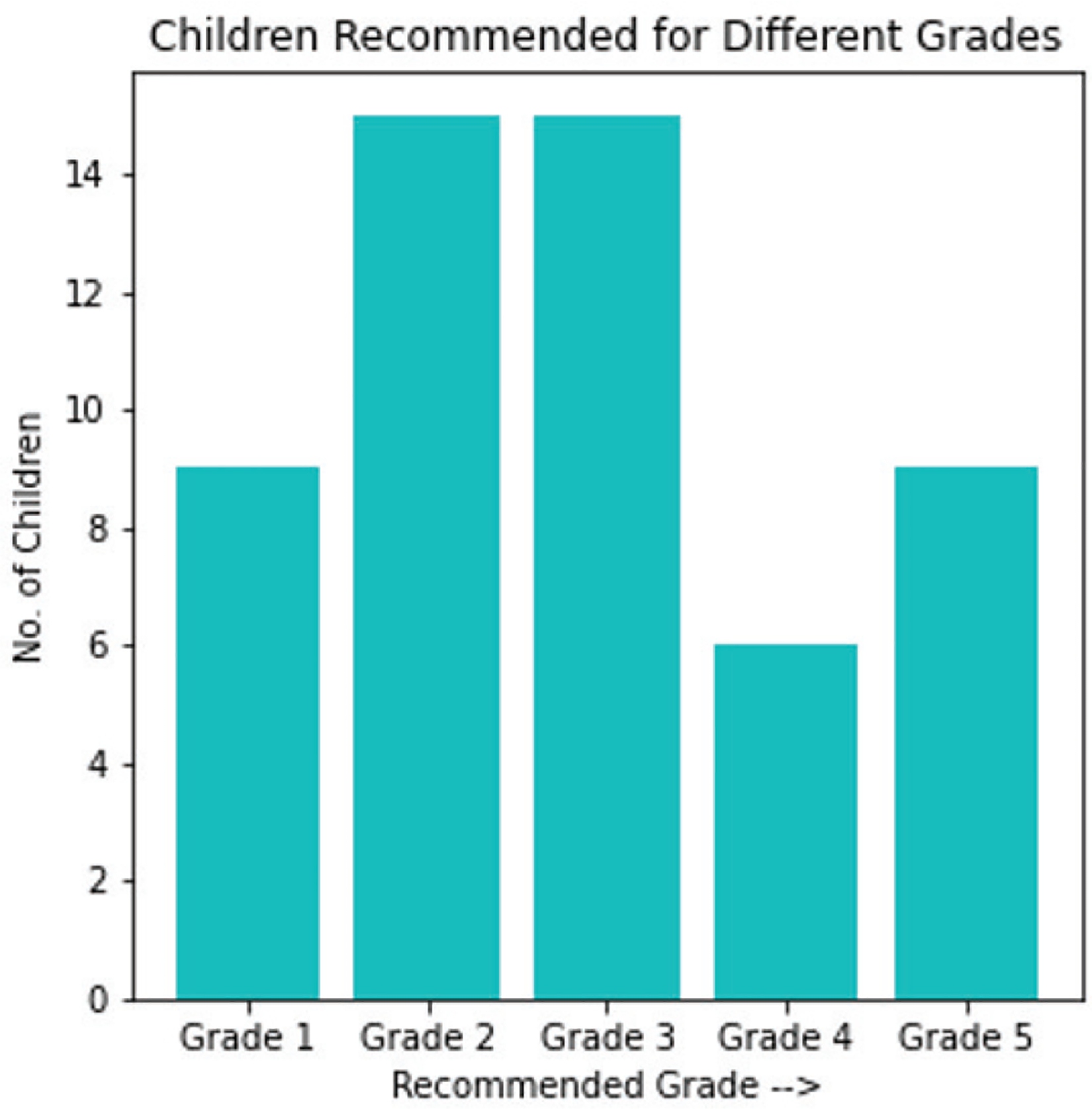
Children recommended for different grades.

To summarize, we contacted 54 Prakash children and administered diagnostic tests that we designed for grades 1 through 5. The tests were presented in the children’s vernacular to ensure that language was not a limiting factor in their performance. The data reveal that most children have math skills at or below grade level 3. This is noteworthy especially given that several of the children are in their mid or late teens and are nominally enrolled in advanced grades in their schools. Figure 6 shows the distribution of recommended grade levels for the participating children. This distribution is in marked contrast to the one shown in figure 2b. It is clear from this discrepancy that the children’s actual level of proficiency lags well behind what their age or grade would suggest. This alarming situation reveals the deficiencies of the current education programs the children are enrolled in and makes a compelling case for more effective educational interventions.

Further studies are needed to identify the factors underlying the problems associated with the current schooling systems for the blind. However, some likely candidates include the poor training of special education teachers and the lack of oversight of schools for the blind. The policy of automatic promotion of children from one grade to the next, even if they do not meet scholastic readiness standards for the higher grade (Right to Education Act, 2018, Government of India), likely contributes to the kind of situation we have witnessed in our results, with putative 12^th^ graders performing below the level of a 4^th^ grader. This policy needs to be rethought.

A caveat that needs to be kept in mind while interpreting these findings is that the diagnostic assessments only addressed math proficiency, which even though important, is only one element of the whole curriculum. It is quite possible that the results would be different depending on the subject assessed. Further studies are needed to comprehensively determine the status of any discrepancies between expected and actual levels of scholastic preparation across other subjects drawn from languages, humanities, and sciences.

### Considerations for designing a new education program

The exercise of administering diagnostic tests to the Prakash children and analyzing the data helps guide our thinking about designing a new education program that can address some of the problems with the current system. The content and organization of this new program must be considered carefully since it has to satisfy multiple constraints, including conformity with state curricula, compatibility with the children’s lingering visual deficits, and the necessity to allow for dynamic, self-paced progression through the program. Additionally, the parents would need to be convinced that the educational intervention is in the child’s best interests to improve his/her prospects of eventually finding a job that can provide them financial independence.

It appears likely that the education program would need to be digitally administered, given the inadequate physical schooling infrastructure. Such a program would have to contend with technological challenges. Although basic phone connectivity has reached most remote places in India, there are still severe bandwidth and stability issues. To be able to provide education remotely, we, therefore, cannot rely exclusively on synchronous online instruction but need to develop more appropriate solutions. One option would be to preload personal digital tablets with specifically designed scholastic material. Several studies, such as Dorouka et. al (2020), Hubber et al. (2016), and Mulet et al. (2019) have assessed the utility of tablet-based interventions in primary education, generally suggesting that tablets are helpful as they are easy to use, enhance interest in the task, and allow students to be independent, thereby encouraging self-education and self-confidence. We believe that combining the tablet-based delivery of scholastic material with regular telephonic check-ins could greatly aid in bridging the educational gap these children face and help bring them to desired academic proficiency levels. An important point to consider in the implementation of such a tablet-based educational program, however, is the sub-par visual status of the students (primarily due to lower than 20/20 acuity (Ganesh et al., 2014), and reduced contrast sensitivity (Kalia et al., 2017)). While tablets are a visual aid, we need to assess how they can be best utilized for low-vision patients and understand what educational apps and content would most benefit the children.

Another aspect worth considering in the design of our education program is that many concepts in mathematics require exposure to various shapes. Visually impaired children are often deficient in these experiences. To remedy this in part, physical aids could synergistically enhance the process of teaching to the visually impaired. Manipulable objects in many forms may assist in learning mathematical concepts and increase comprehension accuracy in visually impaired students (Brawand & Johnson, 2016). Providing physical aids would significantly facilitate the teaching of concepts in geometry.

Finally, the question arises whether, considering the lower-than-normal visual status of many of the Prakash children, including tests of visual function on the digital tablets would be helpful to complement the educational content provided. We believe that the answer is in the affirmative. Assessing Prakash children’s visual status and development over time would allow for a more continuous evaluation of their visual health. Furthermore, it could inform the visual parameters (such as the size, contrast, and color of fonts or other items shown on the tablets) with which the educational contents are being presented. Including such visual assessments may not only serve an essential educational and clinical purpose but could also provide an unprecedented window into the study of visual development by enabling a longitudinally dense tracking of several aspects of visual development, such as visual acuity or color sensitivity profiles.

To conclude, the data we have gathered reveal marked discrepancies between the actual and expected levels of scholastic math preparation in children who have transitioned from blindness to sight. This points to the urgent need to introduce more effective educational approaches for children with visual impairments. We have outlined some considerations relevant for designing a new, potentially digital, education program. Success in this undertaking will be impactful not only for improving educational prospects for children with visual impairments but also for the immensely large population of ‘under-schooled’ children, whose educational preparation is much below age-appropriate levels.
